# Characterization of host substrates of SARS-CoV-2 main protease

**DOI:** 10.3389/fmicb.2023.1251705

**Published:** 2023-08-21

**Authors:** Ivonne Melano, Yan-Chung Lo, Wen-Chi Su

**Affiliations:** ^1^Graduate Institute of Biomedical Sciences, China Medical University, Taichung, Taiwan; ^2^Sinphar Pharmaceutical Co., Ltd., Sinphar Group, Yilan, Taiwan; ^3^International Master’s Program of Biomedical Sciences, China Medical University, Taichung, Taiwan; ^4^Department of Medical Research, China Medical University Hospital, Taichung, Taiwan

**Keywords:** SARS-CoV-2, main protease, substrate, virus-host interaction, virus pathogenesis, viral replication

## Abstract

The main protease (M^pro^) plays a crucial role in coronavirus, as it cleaves viral polyproteins and host cellular proteins to ensure successful replication. In this review, we discuss the preference in the recognition sequence of M^pro^ based on sequence-based studies and structural information and highlight the recent advances in computational and experimental approaches that have aided in discovering novel M^pro^ substrates. In addition, we provide an overview of the current understanding of M^pro^ host substrates and their implications for viral replication and pathogenesis. As M^pro^ has emerged as a promising target for the development of antiviral drugs, further insight into its substrate specificity may contribute to the design of specific inhibitors.

## 1. Introduction

Severe acute respiratory syndrome coronavirus 2 (SARS-CoV-2), the causative agent of the coronavirus disease 2019 (COVID-19) pandemic, is a positive-sense single-stranded RNA virus that utilizes its two cysteine proteases, nsp3/papain-like protease (PL^pro^), and nsp5/3-chymotrypsin-like protease (3CL^pro^), to cleave its polyproteins into functional viral proteins required for virus replication (Koudelka et al., 2021; Sabbah et al., 2021). Nsp3 cleaves three distinct sites of nsp1–nsp4, while nsp5 cleaves 11 distinct sites of nsp5–nsp16; thereby nsp5 is also referred to as the main protease (M^pro^). M^pro^ is a conserved protease in the family Coronaviridae (Ullrich and Nitsche, 2020; Xiong et al., 2021). The mature M^pro^ is a dimeric cysteine protease and its catalytic dyad is formed by His41 and Cys145 (Ullrich and Nitsche, 2020; Hu et al., 2022). Besides viral polyproteins, viral proteases likewise cleave host proteins to hinder host immune responses and promote viral replication (Pablos et al., 2021). In this review, we first address the substrate specificity and further analyze the implication of M^pro^ cleavage on host substrates in various biological processes.

## 2. Substrate specificity of SARS-CoV-2 M^pro^

The substrate specificity of SARS-CoV M^pro^ has been previously investigated. The recombinant protein substrates with saturation mutagenesis at each of the P5 to P3’ positions were used to profile the sequence preference of M^pro^ substrates (Chuck et al., 2010). In addition, the 11 autoproteolytic cleavage site sequences in SARS-CoV-2 pp1ab and host substrates were applied to analyze the sequence logo of the cleavage site. Thus far, the consensus sequence motif of M^pro^ substrates is recognized as (L/F/M)-Q↓(S/A/G/N), where ↓ is the cleavage site. In brief, this motif is composed of a conserved P1 residue Gln flanked by a hydrophobic (Leu, Phe, or Val) at P2 and a small aliphatic amino acid (Ser, Asn, Gly, or Ala) at P1’ positions (Miczi et al., 2020; Koudelka et al., 2021; Moustaqil et al., 2021; Pablos et al., 2021; Zhang et al., 2021). The P1, P2, and P1’ residues are important to determine substrate specificity, whereas the less conserved P3, P4, and P3’ residues increase the recognition and binding stability of the substrates (Hu et al., 2022). P3 and P3’ positions prefer positively charged residues to negatively charged ones (Chuck et al., 2010). Although M^pro^ primarily prefers Gln, it has also been found to recognize non-canonical Met or His at the P1 residue (Koudelka et al., 2021; Pablos et al., 2021). The identification of new substrate sequences can aid in the design of specific inhibitors that can target M^pro^ activity with higher affinity and selectivity.

## 3. Identification of host substrates

Computational and experimental methods are widely used for substrate identification. For computational methods, NetCorona 1.0, a publicly available web server originally designed to predict putative SARS-CoV M^pro^ cleavage sites, has been commonly used for identifying SARS-CoV-2 M^pro^ substrates (with a suggested threshold of 0.5), since the sequence of SARS-CoV-2 M^pro^ shares 96% identity with that of SARS-CoV M^pro^ (Miczi et al., 2020; Zhang et al., 2021; Scott et al., 2022). Another approach is to search for short stretches of homologous human-pathogen protein sequences (SSHHPS) using BLAST analysis, which is based on the principle that the cleavage site sequences found in the viral genome are identical to the cleavage sites on host cell substrates (Miczi et al., 2020). As to experimental methods, a commonly used screening procedure is the liquid chromatography–mass spectrometry (LC–MS)-based terminal amine isotopic labeling of substrates (TAILS) that not only identifies substrates but also their corresponding cleavage sites (Koudelka et al., 2021; Meyer et al., 2021; Pablos et al., 2021). Besides, Moustaqil et al. (2021) screened 71 human innate immune pathway proteins (HIIPs) using the cell-free *Leishmania tarentolae* protein expression system, which allows the direct visualization in SDS-PAGE of the target protein fused to GFP.

**Figure 1:**
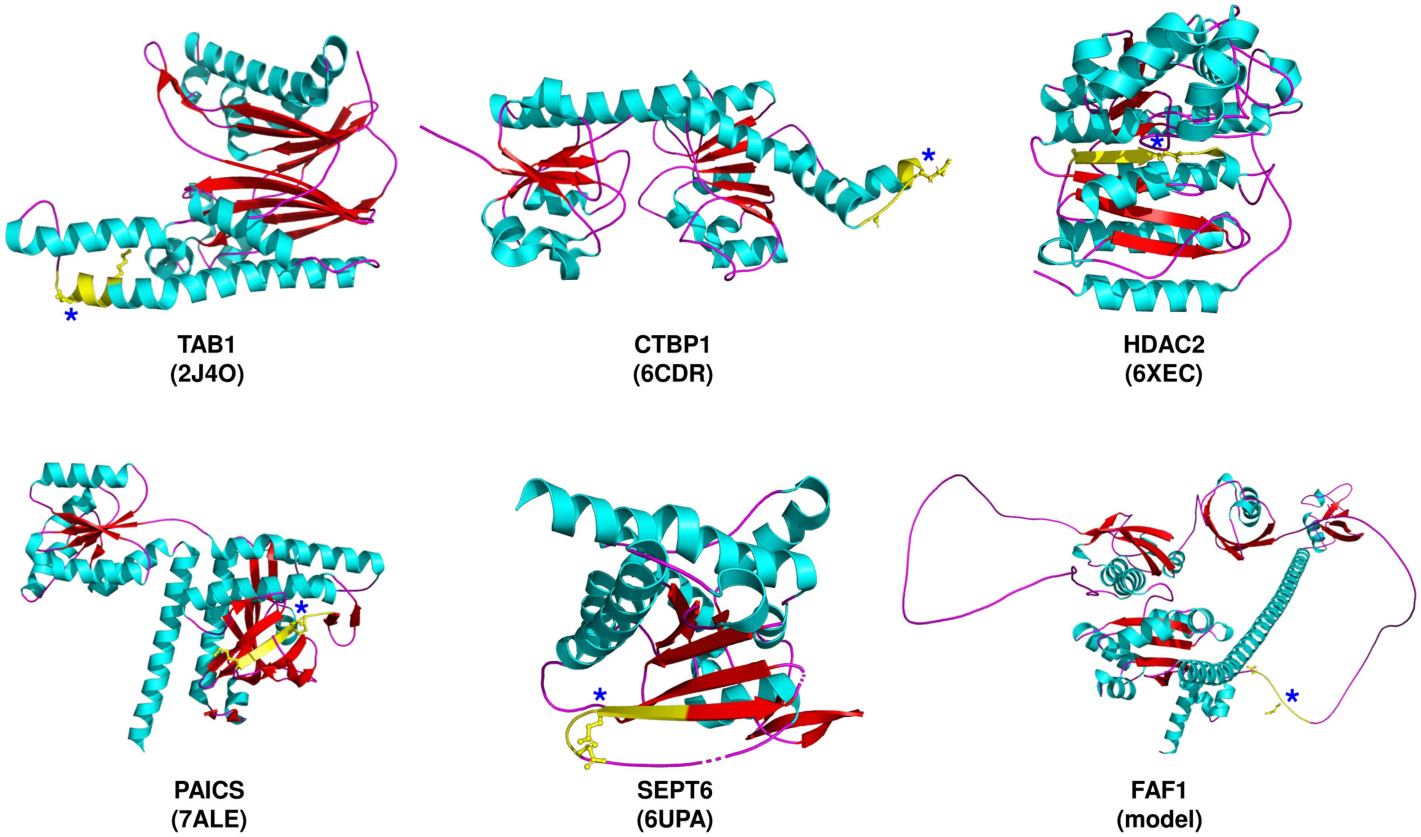
Severe acute respiratory syndrome coronavirus 2 (SARS-CoV-2) M^pro^ cleavage sites in selected target proteins. The proteins are depicted along with the corresponding PDB ID, except for FAF1, which is predicted by AlphaFold. The predicted cleavage sequences (yellow) are shown, with P1 and P5 residues, and an asterisk denoting the P1-Gln residue.

Table 1 lists the host proteins that have been identified as potential substrates for SARS-CoV-2 M^pro^ through computational or experimental methods, and further supported by the detection of cleaved products. Among the identified substrates, five proteins have available structure data in Protein Data Bank (PDB), while the rest were predicted by AlphaFold (Table 1). Through analysis of the structure information, we observed that the cleavage sites are commonly located in loops or loops connected to α-helixes or β-sheets (Figure 1), suggesting that most of the target sequences are accessible to M^pro^. This implies that in addition to the prediction of cleavage sequences, structural analysis is also important for evaluation of the accessibility of putative cleavage sites (Miczi et al., 2020; Moustaqil et al., 2021).

**Table 1:** List of SARS-CoV-2 host substrates.

Gene symbol	Cleavage sequence	Proposed implications of M^pro^ cleavage	Reference	NetCorona score	PDB ID
IRAK1	^453^QSTLQ↓AGL^460^	Decrease cytokines production	Miczi et al. (2020)	0.859	Model
TAB1	^128^KASLQ↓SQL^135^	Inhibit cytokine production	Moustaqil et al. (2021); Pablos et al. (2021)	0.688	2J4O (a.a.16–371)
	^440^TLTLQ↓STN^447^			0.487	
DCP1A	^339^STMMQ↓AVK^346^	Abolish the activity of ISG effector	Song et al. (2023)	0.569	Model
NLRP12	^237^GKLFQ↓GRF^244^	Enhance the production of proinflammatory cytokines and chemokines	Moustaqil et al. (2021)	0.103	Model
	^934^SVVLQ↓ANH^941^			0.902	
SLC25A22	^250^KTRLQ↓SLQ^257^	Decrease immunosuppression	Zhang et al. (2021)	0.938	Model
FAF1	^49^NGILQ↓SEY^56^	Inhibit type 1 interferon signaling	Pablos et al. (2021)	0.224	Model
RPAP1	^14^LLHFQ↓SQF^21^	Divert transcription and translation machineries from host to virus	Pablos et al. (2021)	0.102	Model
	^232^IARLQ↓AMA^239^			0.768	
PTBP1	^148^QAALQ↓AVN^155^	Molecular switch from viral translation to replication	Pablos et al. (2021)	0.445	Model
PNN	^109^KPALQ↓SSV^116^	Transcriptional activation of immune response pathways and induce apoptosis	Meyer et al. (2021)	0.582	Model
CTBP1	^153^GTRVQ↓SVE^160^	Disturb the transcription of host antiviral response genes	Miczi et al. (2020); Scott et al. (2022)	0.946	6CDR
HDAC2	^257^AVVLQ↓CGA^264^	Impairment of ISG expression	Song et al. (2023)	0.328	6XEC (a.a. 1–376)
	^379^GVQMQ↓AIP^386^			0.503	
YAP1	^129^PASLQ↓LGA^136^	Inhibit IRF3 translocation and innate antiviral response	Pablos et al. (2021)	0.243	Model
MAP4K5	^452^ISKLM↓SEN^459^	Block Hippo pathway	Pablos et al. (2021)	NA	Model
CREB1	^205^TTILQ↓YAQ^212^	Regulate transcription of anti-apoptotic genes	Pablos et al. (2021)	0.262	Model
	^225^QVVVQ↓AAS^232^			0.195	
BIRC6	^99^GATLQ↓ASA^106^	Promote apoptosis and autophagy	Zhang et al. (2021)	0.861	N/A
TDP-43	^327^QAALQ↓SSW^334^	Induce cytotoxicity	Yang et al. (2023)	0.378	Model
LGALS8	^154^SSDLQ↓STQ^161^	Escape xenophagy	Pablos et al. (2021)	0.914	Model
FYCO1	^975^LPGLQ↓AQL^982^	Cause incomplete autophagy	Pablos et al. (2021)	0.551	Model
RNF20	^517^SALLQ↓SQS^524^	Stabilizes SREBP1-driven lipid metabolism	Zhang et al. (2021)	0.668	Model
PAICS	^30^KVLLQ↓SKD^37^	promote purine biosynthesis	Meyer et al. (2021)	0.864	7ALE
IRS2	^1118^EAFLQ↓ASQ^1125^	Insulin resistance	Pablos et al. (2021)	0.459	Model
GOLGA3	^446^STKLQ↓AQV^453^	Reconfigure endoplasmic reticulum and Golgi apparatus	Meyer et al. (2021)	0.606	Model
NUP107	^31^RVLLQ↓ASQ^38^	Hijack nuclear pore transport	Meyer et al. (2021); Pablos et al. (2021)	0.569	Model
KPNA3/IMA4	^74^EAILQ↓NAT^81^	Hijack nuclear pore transport	Pablos et al. (2021)	0.495	Model
SEPT2	^336^IARMQ↓AQM^343^	Destabilize filament structure and induce cilia dysfunction	Lee et al. (2023)	0.529	Model
SEPT6	^76^QPGVQ↓LQS^83^	Destabilize filament structure and induce cilia dysfunction	Pablos et al. (2021); Lee et al. (2023)	0.919	6UPA
SEPT9	^216^VSQLQ↓SRL^223^	Destabilize filament structure and induce cilia dysfunction	Pablos et al. (2021); Lee et al. (2023)	0.886	Model

Model: prediction of protein structure by AlphaFold.

N/A, not available.

## 4. Biological functions of substrates

Research on exploring the functional consequences of M^pro^ cleavage on host proteins is still ongoing. It is important to note that host proteins serve multiple functions, and their dysfunction may have implications for more than one biological process. The implications of M^pro^ cleavage, according to published information or the known biological function of the substrates, are discussed below.

### 4.1. Innate immune response

The innate immune system releases inflammatory cytokines and chemokines as an immediate defense against invading pathogens. However, viruses can manipulate the innate immune response to evade the host’s antiviral defenses (Diamond and Kanneganti, 2022). M^pro^ was discovered to cleave interleukin-1 receptor-associated kinase 1 (IRAK1), a kinase involved in the regulation of the innate immune response (Miczi et al., 2020). Several viruses such as porcine epidemic diarrhea virus and borna disease virus 1 target IRAK1 to block IRAK1/TRAF6/NF-κB signaling pathway activation, consequently reducing the expression of the IFN-III subtypes, IFN-λ1, and -λ3 (Zhang et al., 2019; Zheng et al., 2022). Notably, inhibition of IRAK1 using pacritinib had effectively attenuated the pro-inflammatory cytokine release triggered by the GU-rich ssRNA sequence derived from the SARS-CoV-2 spike protein (Campbell et al., 2023). Similarly, the SARS-CoV-2 M^pro^ cleavage of the TAK1 binding protein (TAB1) results in decreased TAB1 protein levels in virus-infected cells and is proposed to inhibit cytokine production by disrupting the interaction between TAB1 and the transforming growth factor-β-activated kinase 1 (TAK1), which is necessary for constitutive activation of NF-κB (Jackson-Bernitsas et al., 2007; Moustaqil et al., 2021; Pablos et al., 2021). mRNA-decapping enzyme 1A (DCP1A), one of the interferon-stimulated genes (ISGs), was recently identified as an M^pro^ substrate (Song et al., 2023). Cleavage of DCP1A by porcine deltacoronavirus M^pro^ has been demonstrated to decrease antiviral activity (Zhu et al., 2020). It is conceivable that SARS-CoV-2 M^pro^ cleaves IRAK1, TAB1, and DCP1A to disturb the production of pro-inflammatory cytokines and attenuate the immune defense (Miczi et al., 2020).

On the other hand, hyperinflammation, characterized by cytokine storm, is a significant contributor to severe cases of COVID-19 (Diamond and Kanneganti, 2022). SARS-CoV-2 M^pro^ specifically cleaved Nod-like receptor protein 12 (NLRP12), as evidenced by significant reductions of NLRP12 protein levels in SARS-CoV-2 infected cells (Moustaqil et al., 2021). Its cleavage is proposed to enhance pro-inflammatory cytokine and chemokine production via NF-κB signaling, and perturb the NLRP3 inflammasome assembly to trigger the cleavage of pro-caspase-1, thereby enhancing the release of IL-1β, all associated with the hyperinflammation observed in severe COVID-19. Another ISG cleaved by M^pro^ is the solute carrier family 25 member 22 (SLC25A22; Zhang et al., 2021). Knockout of SLC25A22, a mitochondrial glutamate carrier, has been associated with decreased immunosuppressive function in colorectal cancer (Yoo et al., 2020; Zhou et al., 2021), implying its involvement in immune response activation.

Fas-associated factor 1 (FAF1) is a positive regulator of type I interferon (IFN) signaling and is involved in the activation of the Fas-mediated pathway of apoptosis. However, there are contrasting results on the role of FAF1 in regulating the antiviral immune response. FAF1 is suggested to reduce virus-induced type I IFN activation by inhibiting nuclear translocation of the transcription factor IRF3 (Song et al., 2016). In contrast, FAF1 is hypothesized to bind competitively to NLRX1 to free the mitochondrial antiviral signaling protein (MAVS) upon RNA virus infection, which subsequently interacts with the retinoic acid-inducible gene (RIG)-I to initiate type I IFN signaling (Kim et al., 2017). Furthermore, virus infection is postulated to prevent aggregation of FAF1, which inhibits FAF1-dependent suppression of MAVS and then activates antiviral immunity (Dai et al., 2018). More studies are needed to confirm the role of FAF1 cleavage in virus infection.

### 4.2. Transcription and translation

Viruses can affect host gene expression at the transcriptional level. In addition, since viruses lack functional ribosomes, they attempt to usurp the host’s translational apparatus by competing with cellular mRNA to achieve successful replication. For instance, RNA polymerase II-associated protein 1 (RPAP1), which is crucial to bridging RNA polymerase II with gene-enhancer elements to increase transcription, and the polypyrimidine tract-binding protein (PTBP1), essential for pre-mRNA splicing and mRNA export, are both cleaved by M^pro^. Proteolysis of PTBP1 after SARS-CoV-2 infection leads to the redistribution of PTBP1 from the nucleus to the cytoplasm (Pablos et al., 2021). In polioviruses, proteolysis of PTBP1 is speculated to switch viral translation to replication (Back et al., 2002). Thus, M^pro^ might target RPAP1 and PTBP1 to divert transcription and translation machineries from host to virus.

Pinin (PNN), a multifunctional nuclear phosphoprotein involved in the regulation of transcription and alternative RNA splicing, has also been identified as a substrate of M^pro^ (Meyer et al., 2021). Depletion of PNN has been demonstrated to result in apoptosis *in vitro* and early lethality *in vivo* (Leu et al., 2012). Furthermore, PNN binds to the transcriptional co-repressor C-terminal binding protein 1 (CTBP1). The interaction of PNN and CTBP1 alters CTBP1 silencing function (Alpatov et al., 2004). The overlapping pathways enriched in PNN-KD and CTBP1-KD cells include the TNFα-induced canonical NFκB signaling pathway and the IFN response pathway (Zhang et al., 2016). CTBP1-mutated neuronal cells were more susceptible to West Nile virus than control cells, consistent with the lower expression of IFN-response genes in CTBP1-mutated cells (Vijayalingam et al., 2020). Cleavage of PNN and CTBP1 by M^pro^ is suggested to alter the transcription of host antiviral response genes and induce apoptosis (Miczi et al., 2020; Meyer et al., 2021). Furthermore, M^pro^ cleaves Histone deacetylase 2 (HDAC2), which primarily regulates gene transcription by modifying histones and is also required for ISG transcriptional elongation (Chang et al., 2004). In consequence, the cleavage of HDAC2 by M^pro^ results in the impairment of ISG expression (Song et al., 2023).

Yes-associated protein 1 (YAP1), a transcriptional co-activator, participates in Hippo pathway. Since YAP negatively regulated an antiviral immune response via inhibiting the translocation of IRF3 to the nucleus, cleavage of YAP1 is presumed to enhance innate immunity (Wang et al., 2017). The kinase activity of mitogen-activated kinase-kinase-kinase-kinase 5 (MAP4K5), another Hippo pathway regulator, can be inactivated by M^pro^ cleavage. cAMP response element binding protein 1 (CREB1) is a transcription factor that dimerizes with ATF1 to regulate the transcription of anti-apoptotic and cell proliferation genes. Besides, CREB1 binds YAP1 and forms a positive feedback loop with each other (Chen et al., 2018). M^pro^ cleavages of YAP1, MAP4K5, and CREB1 indicate that SARS-CoV-2 can hijack the Hippo-YAP signaling pathway (Pablos et al., 2021) for mediating a variety of cellular processes, including cell proliferation, differentiation, apoptosis, and immune response.

### 4.3. Apoptosis and autophagy

To maintain homeostasis, cells undergo two types of programmed cell death (PCD)-apoptosis and autophagy (Kennedy, 2015). Inhibition of these PCDs by SARS-CoV-2 aids the virus to avoid elimination in the cells and ensure viable cells for viral replication, while induction may benefit the virus by the regulation of immune response and virus release (Li et al., 2020, 2021, 2022). Moreover, SARS-CoV-2 exploits autophagy to prevent virus degradation (Chen et al., 2020). Several proteins involved in apoptosis and autophagy have been identified to be targeted by M^pro^.

Baculoviral IAP repeat-containing protein 6 (BIRC6) functions as an inhibitor of apoptosis and autophagy by ubiquitinating pro-apoptotic factors and LC3B, leading to their proteasomal degradation (Ehrmann et al., 2022). M^pro^ cleavage of BIRC6 may promote apoptosis and autophagy, in line with the induction of apoptosis and autophagy upon SARS-CoV-2 infection (Li et al., 2020, 2021). Transactive response DNA binding protein 43 kDa (TDP-43) is critical in RNA regulation, including the expression of viral RNA (reviewed in Rahic et al., 2023). Cleavage of TDP-43 by M^pro^ induced cytotoxicity in neurons, which could contribute to the pathogenicity of SARS-CoV-2 in the nervous system (Yang et al., 2023).

Galectin-8 (LGALS8) is involved in the regulation of immune responses and directly binds to Spike S1 glycans and the autophagy adaptor NDP52 (Pablos et al., 2021). LGALS8 is proposed to sense the glycosylated Spike S1 protein and activate xenophagy, a type of selective autophagy targeting invading pathogens to lysosomes, to reduce SARS-CoV-2 infection (Pablos et al., 2021). Furthermore, the autophagy adaptor protein FYVE and the coiled coil domain containing 1 (FYCO1) has been identified as a candidate COVID-19 susceptibility and severity gene and is believed to be the key mediator that connects double-membrane vesicles (the main site of coronavirus replication) from the endoplasmic reticulum to the microtubule network in host cells (Reggiori et al., 2011; Parkinson et al., 2020; Lee et al., 2021; Jahanafrooz et al., 2022). The elimination of FYCO1 resulted in the accumulation of early autophagosomes (Pankiv et al., 2010). M^pro^ cleavage of LGALS8 and FYCO1 possibly enables SARS-CoV-2 to escape antiviral xenophagy (Pablos et al., 2021) and induce incomplete autophagy.

### 4.4. Cell metabolism

SARS-CoV-2 infection alters host cell metabolism (Andrade Silva et al., 2021; Mullen et al., 2021). In fact, proteins that play roles in cell metabolism were found to be substrates of M^pro^. Cleavage of Ring finger protein 20 (RNF20) destabilizes the RNF20/RNF40 complex, which is essential for their ubiquitin E3 ligase activity. As a result, this blocks the degradation of the sterol regulatory element binding protein 1 (SREBP1), and subsequently increasing the lipid metabolism for promoting SARS-CoV-2 replication (Zhang et al., 2021).

Phosphoribosylaminoimidazole succinocarboxamide synthetase (PAICS), a *de novo* purine biosynthetic enzyme was previously identified to be crucial in influenza virus replication (Karlas et al., 2010; Generous et al., 2014). PAICS is proposed to be a candidate for a noncanonical route for SARS-CoV-2 infection in human placentas (Constantino et al., 2021). SARS-CoV-2 infection has been reported to promote *de novo* purine synthesis through nsp9 (Qin et al., 2022). Silencing of PAICS reduced virus titers (~10-fold), suggesting that cleavage of PAICS by M^pro^ results in altered function of PAICS (Meyer et al., 2021), which may influence the *de novo* purine synthesis.

Insulin receptor substrate 2 (IRS2) regulates insulin signaling and the control of glucose homeostasis. Hepatitis C virus infection downregulates IRS2 expression by upregulating the suppressor of cytokine signaling (SOCS) and by activating the mTOR/S6K1 signaling pathway, resulting in insulin resistance (Kawaguchi et al., 2004; Pazienza et al., 2007; Bose et al., 2012). Notably, new-onset hyperglycemia has been associated with SARS-CoV-2 because non-diabetic COVID-19 patients were found to have increased risk of insulin resistance (Chen et al., 2021; Wihandani et al., 2023), which may be associated with M^pro^ cleavage of IRS2 (Pablos et al., 2021).

### 4.5. Intracellular transport and cytoskeleton

The intracellular transport system and cytoskeletons are essential for viral infections, particularly for transporting viral components to specific subcellular compartment sites of translation, replication, and secretion. The Golgi apparatus is an integral component of the viral life cycle. SARS-CoV-2 remodels the Golgi structure for viral release, hence, M^pro^ cleavage of Golgin subfamily A member 3 (GOLGA3), which is involved in the organization of the Golgi apparatus and its associated vesicles (Meyer et al., 2021; Pablos et al., 2021), may also be linked to this modulation (Zhang et al., 2022). Moreover, GOLGA3 has been associated with COVID-19 and has been identified to interact with nsp13 (Gordon et al., 2020; Deng et al., 2021). M^pro^ cleavage of GOLGA3 may play a role in reconfiguring the endoplasmic reticulum to facilitate Golgi trafficking during virus assembly.

Although RNA viruses replicate in the cytoplasm, they also exploit the nucleocytoplasmic trafficking system to inhibit the host immune response (Sajidah et al., 2021), which may explain why SARS-CoV-2 M^pro^ cleaves the nuclear pore complex 107 kDa subunit (NUP107) and Importin subunit alpha-4 (IMA4), which are both important members of nuclear pore transport (Pablos et al., 2021). IMA4, also known as karyopherin subunit alpha-3 (KPNA3), has been shown to be targeted by the Japanese encephalitis virus NS5 protein to hinder the nuclear import of its cargo molecules IFN regulatory factor 3 and NF-κB, thereby subsequently inhibiting type 1 IFN production (Ye et al., 2017).

Septin (SEPT) is recognized as a component of the cytoskeleton (Mostowy and Cossart, 2012). Septin polymerizes into filaments at the cell cortex or in association with other cytoskeletal proteins, such as actin or microtubules. M^pro^ cleaves several septin proteins, including SEPT2, SEPT6, and SEPT9, to affect the septin complex, causing an unstable filament structure and inducing cilia dysfunction (Lee et al., 2023).

## 5. Discussion

With the help of computational and experimental methods, scientists have gained valuable insights into the substrates of M^pro^. NetCorona analysis is widely used for substrate prediction. Intriguingly, some of the identified substrates have low NetCorona scores (Table 1), implying other issues should be considered. Further information, like binding affinity, may improve the original algorithm. The steric effects on substrate specificity also play an important role for the assessment. Notably, the cleavage sites of HDAC2 and PAICS are buried in the structure, warranting further study regarding the mechanism of M^pro^ cleavage of these two proteins. Deep learning of sequenced-based prediction and structural analysis can likewise improve the accuracy of prediction.

Identification of viral host substrates helps determine specific virus-host interactions, including the cellular pathways involved, and the mechanisms of viral replication and pathogenesis. Consequently, researchers can gain valuable insights into how viruses cause diseases and develop strategies to control or treat viral infections. After COVID-19 infection, certain individuals developed post-acute sequelae of SARS-CoV-2 infection (PASC), known as long COVID. The persistence of viral RNA or proteins for weeks in these patients implies the presence of an impaired immune response. Exploring the potential role of M^pro^ in this aspect would be valuable. Besides, identifying the specific sequences of host substrates targeted by M^pro^ can have significant implications in developing peptidomimetic protease inhibitors. Discovering new substrate sequences can enhance the design of effective antiviral strategies. Continued research is essential to improve our understanding of M^pro^ function and develop potent antiviral therapies against coronaviruses.

## Author contributions

W-CS conceived and supervised the review topic. IM, Y-CL, and W-CS participated in the writing and preparation of the manuscript. All authors contributed to the article and approved the submitted version.

## Funding

This work is supported by the grant (MOST 111-2320-B-039-060) from the National Science and Technology Council, Taiwan, and the grant (CMU111-MF-29) from China Medical University.

## Conflict of interest

Y-CL was employed by the company Sinphar Pharmaceutical Co, Ltd.

The remaining authors declare that the research was conducted in the absence of any commercial or financial relationships that could be construed as a potential conflict of interest.

## Publisher’s note

All claims expressed in this article are solely those of the authors and do not necessarily represent those of their affiliated organizations, or those of the publisher, the editors and the reviewers. Any product that may be evaluated in this article, or claim that may be made by its manufacturer, is not guaranteed or endorsed by the publisher.

## References

[ref1] Alpatov R. Munguba G. C. Caton P. Joo J. H. Shi Y. Shi Y.. (2004). Nuclear speckle-associated protein Pnn/Drs binds to the transcriptional corepressor Ctbp and relieves Ctbp-mediated repression of the E-cadherin gene. Mol. Cell. Biol. 24, 10223–10235. doi: 10.1128/MCB.24.23.10223-10235.2004, PMID: 15542832 PMC529029

[ref2] Andrade Silva M. Da Silva A. Do Amaral M. A. Fragas M. G. Câmara N. O. S. (2021). Metabolic alterations in Sars-Cov-2 infection and its implication in kidney dysfunction. Front. Physiol. 12:624698. doi: 10.3389/fphys.2021.624698, PMID: 33716771 PMC7947848

[ref3] Back S. H. Kim Y. K. Kim W. J. Cho S. Oh H. R. Kim J.-E.. (2002). Translation of polioviral mRNA is inhibited by cleavage of polypyrimidine tract-binding proteins executed by polioviral 3cPro. J. Virol. 76, 2529–2542. doi: 10.1128/jvi.76.5.2529-2542.2002, PMID: 11836431 PMC135932

[ref4] Bose S. K. Shrivastava S. Meyer K. Ray R. B. Ray R. (2012). Hepatitis C virus activates the Mtor/S6k1 signaling pathway in inhibiting Irs-1 function for insulin resistance. J. Virol. 86, 6315–6322. doi: 10.1128/JVI.00050-12, PMID: 22457523 PMC3372214

[ref5] Campbell G. R. Rawat P. Spector S. A. (2023). Pacritinib inhibition of Irak1 blocks aberrant Tlr8 signalling by Sars-Cov-2 and Hiv-1-derived RNA. J. Innate Immun. 15, 96–106. doi: 10.1159/000525292, PMID: 35785771 PMC10643889

[ref6] Chang H. M. Paulson M. Holko M. Rice C. M. Williams B. R. Marie I.. (2004). Induction of interferon-stimulated gene expression and antiviral responses require protein deacetylase activity. Proc. Natl. Acad. Sci. U. S. A. 101, 9578–9583. doi: 10.1073/pnas.0400567101, PMID: 15210966 PMC470717

[ref7] Chen L. Feng P. Peng A. Qiu X. Zhu X. He S.. (2018). Camp response element-binding protein and yes-associated protein form a feedback loop that promotes neurite outgrowth. J. Cell. Mol. Med. 22, 374–381. doi: 10.1111/jcmm.13324, PMID: 28857442 PMC5742726

[ref8] Chen S.-W. Himeno M. Koui Y. Sugiyama M. Nishitsuji H. Mizokami M.. (2020). Modulation of hepatitis B virus infection by epidermal growth factor secreted from liver sinusoidal endothelial cells. Sci. Rep. 10:14349. doi: 10.1038/s41598-020-71453-5, PMID: 32873852 PMC7462976

[ref9] Chen M. Zhu B. Chen D. Hu X. Xu X. Shen W. J.. (2021). Covid-19 may increase the risk of insulin resistance in adult patients without diabetes: a 6-month prospective study. Endocr. Pract. 27, 834–841. doi: 10.1016/j.eprac.2021.04.004, PMID: 33887468 PMC8054613

[ref10] Chuck C. P. Chong L. T. Chen C. Chow H. F. Wan D. C. Wong K. B. (2010). Profiling of substrate specificity of Sars-Cov 3cl. PLoS One 5:E13197. doi: 10.1371/journal.pone.0013197, PMID: 20949131 PMC2950840

[ref11] Constantino F. B. Cury S. S. Nogueira C. R. Carvalho R. F. Justulin L. A. (2021). Prediction of non-canonical routes for Sars-Cov-2 infection in human placenta cells. Front. Mol. Biosci. 8:614728. doi: 10.3389/fmolb.2021.614728, PMID: 34820418 PMC8606885

[ref12] Dai T. Wu L. Wang S. Wang J. Xie F. Zhang Z.. (2018). Faf1 regulates antiviral immunity by inhibiting mavs but is antagonized by phosphorylation upon viral infection. Cell Host Microbe 24, 776–790.E5. doi: 10.1016/j.chom.2018.10.006, PMID: 30472208

[ref13] Deng H. Yan X. Yuan L. (2021). Human genetic basis of coronavirus disease 2019. Signal Transduct. Target. Ther. 6:344. doi: 10.1038/s41392-021-00736-8, PMID: 34545062 PMC8450706

[ref14] Diamond M. S. Kanneganti T.-D. (2022). Innate immunity: the first line of defense against Sars-Cov-2. Nat. Immunol. 23, 165–176. doi: 10.1038/s41590-021-01091-0, PMID: 35105981 PMC8935980

[ref15] Ehrmann J. F. Grabarczyk D. B. Heinke M. Deszcz L. Kurzbauer R. Hudecz O.. (2022). Structural basis of how the Birc6/SMAC complex regulates apoptosis and autophagy. Biorxiv [Preprint]. doi: 10.1126/science.ade8873

[ref16] Generous A. Thorson M. Barcus J. Jacher J. Busch M. Sleister H. (2014). Identification of putative interactions between swine and human influenza a virus nucleoprotein and human host proteins. Virol. J. 11:228. doi: 10.1186/s12985-014-0228-6, PMID: 25547032 PMC4297426

[ref17] Gordon D. E. Jang G. M. Bouhaddou M. Xu J. Obernier K. White K. M.. (2020). A Sars-Cov-2 protein interaction map reveals targets for drug repurposing. Nature 583, 459–468. doi: 10.1038/s41586-020-2286-9, PMID: 32353859 PMC7431030

[ref18] Hu Q. Xiong Y. Zhu G. H. Zhang Y. N. Zhang Y. W. Huang P.. (2022). The Sars-Cov-2 main protease (M(pro)): structure, function, and emerging therapies for Covid-19. Medcomm 3:E151. doi: 10.1002/mco2.151 35845352 PMC9283855

[ref19] Jackson-Bernitsas D. G. Ichikawa H. Takada Y. Myers J. N. Lin X. L. Darnay B. G.. (2007). Evidence that Tnf-Tnfr1-Tradd-Traf2-rip-Tak1-Ikk pathway mediates constitutive Nf-Κb activation and proliferation in human head and neck squamous cell carcinoma. Oncogene 26, 1385–1397. doi: 10.1038/sj.onc.1209945, PMID: 16953224

[ref20] Jahanafrooz Z. Chen Z. Bao J. Li H. Lipworth L. Guo X. (2022). An overview of human proteins and genes involved in Sars-Cov-2 infection. Gene 808:145963. doi: 10.1016/j.gene.2021.145963, PMID: 34530086 PMC8437745

[ref21] Karlas A. Machuy N. Shin Y. Pleissner K.-P. Artarini A. Heuer D.. (2010). Genome-wide RNAi screen identifies human host factors crucial for influenza virus replication. Nature 463, 818–822. doi: 10.1038/nature08760, PMID: 20081832

[ref22] Kawaguchi T. Yoshida T. Harada M. Hisamoto T. Nagao Y. Ide T.. (2004). Hepatitis C virus down-regulates insulin receptor substrates 1 and 2 through up-regulation of suppressor of cytokine signaling 3. Am. J. Pathol. 165, 1499–1508. doi: 10.1016/S0002-9440(10)63408-6, PMID: 15509521 PMC1618659

[ref23] Kennedy P. G. E. (2015). Viruses, apoptosis, and neuroinflammation—a double-edged sword. J. Neuro-Oncol. 21, 1–7. doi: 10.1007/s13365-014-0306-y, PMID: 25604493

[ref24] Kim J.-H. Park M.-E. Nikapitiya C. Kim T.-H. Uddin M. B. Lee H.-C.. (2017). Fas-associated factor-1 positively regulates type i interferon response to RNA virus infection by targeting Nlrx1. PLoS Pathog. 13:E1006398. doi: 10.1371/journal.ppat.1006398, PMID: 28542569 PMC5456407

[ref25] Koudelka T. Boger J. Henkel A. Schonherr R. Krantz S. Fuchs S.. (2021). N-terminomics for the identification of in vitro substrates and cleavage site specificity of the Sars-Cov-2 main protease. Proteomics 21:2000246. doi: 10.1002/pmic.202000246 33111431 PMC7645863

[ref26] Lee A. R. Kweon Y. C. Lee S. M. Park C. Y. (2023). Human coronavirus 3cl proteases cleave septins and disrupt hedgehog signaling. J. Med. Virol. 95:E28618. doi: 10.1002/jmv.28618 36840410

[ref27] Lee J.-W. Lee I.-H. Sato T. Kong S. W. Iimura T. (2021). Genetic variation analyses indicate conserved Sars-Cov-2–host interaction and varied genetic adaptation in immune response factors in modern human evolution. Develop. Growth Differ. 63, 219–227. doi: 10.1111/dgd.12717, PMID: 33595856 PMC8013644

[ref28] Leu S. Lin Y.-M. Wu C.-H. Ouyang P. (2012). Loss of PNN expression results in mouse early embryonic lethality and cellular apoptosis through Srsf1-mediated alternative expression of Bcl-Xs and Icad. J. Cell Sci. 125, 3164–3172. doi: 10.1242/jcs.100859, PMID: 22454513

[ref29] Li F. Li J. Wang P. H. Yang N. Huang J. Ou J.. (2021). Sars-Cov-2 spike promotes inflammation and apoptosis through autophagy by Ros-suppressed Pi3k/Akt/Mtor signaling. Biochim. Biophys. Acta Mol. basis Dis. 1867:166260. doi: 10.1016/j.bbadis.2021.166260, PMID: 34461258 PMC8390448

[ref30] Li S. Zhang Y. Guan Z. Li H. Ye M. Chen X.. (2020). Sars-Cov-2 triggers inflammatory responses and cell death through Caspase-8 activation. Signal Transduct. Target. Ther. 5:235. doi: 10.1038/s41392-020-00334-0, PMID: 33037188 PMC7545816

[ref31] Li X. Zhang Z. Wang Z. Gutiérrez-Castrellón P. Shi H. (2022). Cell deaths: involvement in the pathogenesis and intervention therapy of Covid-19. Signal Transduct. Target. Ther. 7:186. doi: 10.1038/s41392-022-01043-6, PMID: 35697684 PMC9189267

[ref32] Meyer B. Chiaravalli J. Gellenoncourt S. Brownridge P. Bryne D. P. Daly L. A.. (2021). Characterising proteolysis during Sars-Cov-2 infection identifies viral cleavage sites and cellular targets with therapeutic potential. Nat. Commun. 12:5553. doi: 10.1038/s41467-021-25796-w, PMID: 34548480 PMC8455558

[ref33] Miczi M. Golda M. Kunkli B. Nagy T. Tozser J. Motyan J. A. (2020). Identification of host cellular protein substrates of Sars-Cov-2 main protease. Int. J. Mol. Sci. 21:9523. doi: 10.3390/ijms21249523, PMID: 33333742 PMC7765187

[ref34] Mostowy S. Cossart P. (2012). Septins: the fourth component of the cytoskeleton. Nat. Rev. Mol. Cell Biol. 13, 183–194. doi: 10.1038/nrm3284, PMID: 22314400

[ref35] Moustaqil M. Ollivier E. Chiu H. P. Van Tol S. Rudolffi-Soto P. Stevens C.. (2021). Sars-Cov-2 proteases Plpro and 3clpro cleave Irf3 and critical modulators of inflammatory pathways (NLRP12 and TAB1): implications for disease presentation across species. Emerg. Microbes Infect. 10, 178–195. doi: 10.1080/22221751.2020.1870414, PMID: 33372854 PMC7850364

[ref36] Mullen P. J. Garcia G. Purkayastha A. Matulionis N. Schmid E. W. Momcilovic M.. (2021). Sars-Cov-2 infection rewires host cell metabolism and is potentially susceptible to Mtorc1 inhibition. Nat. Commun. 12:1876. doi: 10.1038/s41467-021-22166-4, PMID: 33767183 PMC7994801

[ref37] Pablos I. Machado Y. De Jesus H. C. R. Mohamud Y. Kappelhoff R. Lindskog C.. (2021). Mechanistic insights into Covid-19 by global analysis of the Sars-Cov-2 3cl(pro) substrate degradome. Cell Rep. 37:109892. doi: 10.1016/j.celrep.2021.109892, PMID: 34672947 PMC8501228

[ref38] Pankiv S. Alemu E. A. Brech A. Bruun J. A. Lamark T. Overvatn A.. (2010). Fyco1 is a Rab7 effector that binds to Lc3 and Pi3p to mediate microtubule plus end-directed vesicle transport. J. Cell Biol. 188, 253–269. doi: 10.1083/jcb.200907015, PMID: 20100911 PMC2812517

[ref39] Parkinson N. Rodgers N. Head Fourman M. Wang B. Zechner M. Swets M. C.. (2020). Dynamic data-driven meta-analysis for prioritisation of host genes implicated in COVID-19. Sci. Rep. 10:22303. doi: 10.1038/s41598-020-79033-3, PMID: 33339864 PMC7749145

[ref40] Pazienza V. Clément S. Pugnale P. Conzelman S. Foti M. Mangia A.. (2007). The hepatitis C virus core protein of genotypes 3a and 1b downregulates insulin receptor substrate 1 through genotype-specific mechanisms. Hepatology 45, 1164–1171. doi: 10.1002/hep.21634, PMID: 17465001

[ref41] Qin C. Rao Y. Yuan H. Wang T. Y. Zhao J. Espinosa B.. (2022). Sars-Cov-2 couples evasion of inflammatory response to activated nucleotide synthesis. Proc. Natl. Acad. Sci. U. S. A. 119:E2122897119. doi: 10.1073/pnas.2122897119, PMID: 35700355 PMC9245715

[ref42] Rahic Z. Buratti E. Cappelli S. (2023). Reviewing the potential links between viral infections and Tdp-43 proteinopathies. Int. J. Mol. Sci. 24:1581. doi: 10.3390/ijms24021581, PMID: 36675095 PMC9867397

[ref43] Reggiori F. De Haan C. A. M. Molinari M. (2011). Unconventional use of Lc3 by coronaviruses through the alleged subversion of the erad tuning pathway. Viruses 3, 1610–1623. doi: 10.3390/v3091610, PMID: 21994798 PMC3187687

[ref44] Sabbah D. A. Hajjo R. Bardaweel S. K. Zhong H. A. (2021). An updated review on Sars-Cov-2 main proteinase (M(pro)): protein structure and small-molecule inhibitors. Curr. Top. Med. Chem. 21, 442–460. doi: 10.2174/1568026620666201207095117, PMID: 33292134

[ref45] Sajidah E. S. Lim K. Wong R. W. (2021). How Sars-Cov-2 and other viruses build an invasion route to hijack the host nucleocytoplasmic trafficking system. Cells 10:1424. doi: 10.3390/cells10061424, PMID: 34200500 PMC8230057

[ref46] Scott B. M. Lacasse V. Blom D. G. Tonner P. D. Blom N. S. (2022). Predicted coronavirus Nsp5 protease cleavage sites in the human proteome. BMC Genom Data 23:25. doi: 10.1186/s12863-022-01044-y, PMID: 35379171 PMC8977440

[ref47] Song S. Lee J. J. Kim H. J. Lee J. Y. Chang J. Lee K. J. (2016). FAS-associated factor 1 negatively regulates the antiviral immune response by inhibiting translocation of interferon regulatory factor 3 to the nucleus. Mol. Cell. Biol. 36, 1136–1151. doi: 10.1128/MCB.00744-15, PMID: 26811330 PMC4800795

[ref48] Song L. Wang D. Abbas G. Li M. Cui M. Wang J.. (2023). The main protease of Sars-Cov-2 cleaves histone deacetylases and Dcp1a, attenuating the immune defense of the interferon-stimulated genes. J. Biol. Chem. 299:102990. doi: 10.1016/j.jbc.2023.102990, PMID: 36758802 PMC9907797

[ref49] Ullrich S. Nitsche C. (2020). The Sars-Cov-2 main protease as drug target. Bioorg. Med. Chem. Lett. 30:127377. doi: 10.1016/j.bmcl.2020.127377, PMID: 32738988 PMC7331567

[ref50] Vijayalingam S. Ezekiel U. R. Xu F. Subramanian T. Geerling E. Hoelscher B.. (2020). Human IPSC-derived neuronal cells from Ctbp1-mutated patients reveal altered expression of neurodevelopmental gene networks. Front. Neurosci. 14:562292. doi: 10.3389/fnins.2020.562292 33192249 PMC7653094

[ref51] Wang S. Xie F. Chu F. Zhang Z. Yang B. Dai T.. (2017). Yap antagonizes innate antiviral immunity and is targeted for lysosomal degradation through Ikkɛ-mediated phosphorylation. Nat. Immunol. 18, 733–743. doi: 10.1038/ni.3744, PMID: 28481329

[ref52] Wihandani D. M. Purwanta M. L. A. Mulyani W. R. W. Putra I. Supadmanaba I. G. P. (2023). New-onset diabetes in Covid-19: the molecular pathogenesis. Biomedicine 13, 3–12. doi: 10.37796/2211-8039.1389, PMID: 37168726 PMC10166251

[ref53] Xiong M. Su H. Zhao W. Xie H. Shao Q. Xu Y. (2021). What coronavirus 3c-like protease tells us: from structure, substrate selectivity, to inhibitor design. Med. Res. Rev. 41, 1965–1998. doi: 10.1002/med.21783, PMID: 33460213 PMC8014231

[ref54] Yang J. Li Y. Wang S. Li H. Zhang L. Zhang H.. (2023). The Sars-Cov-2 main protease induces neurotoxic Tdp-43 cleavage and aggregates. Signal Transduct. Target. Ther. 8:109. doi: 10.1038/s41392-023-01386-8, PMID: 36894543 PMC9998009

[ref55] Ye J. Chen Z. Li Y. Zhao Z. He W. Zohaib A.. (2017). Japanese encephalitis virus Ns5 inhibits type I interferon (IFN) production by blocking the nuclear translocation of IFN regulatory factor 3 and Nf-Κb. J. Virol. 91, E00039–E00017. doi: 10.1128/JVI.00039-17 28179530 PMC5375679

[ref56] Yoo H. C. Yu Y. C. Sung Y. Han J. M. (2020). Glutamine reliance in cell metabolism. Exp. Mol. Med. 52, 1496–1516. doi: 10.1038/s12276-020-00504-8, PMID: 32943735 PMC8080614

[ref57] Zhang X. Guo Y. Xu X. Tang T. Sun L. Wang H.. (2019). Mir-146a promotes Borna disease virus 1 replication through Irak1/Traf6/Nf-Κb signaling pathway. Virus Res. 271:197671. doi: 10.1016/j.virusres.2019.197671, PMID: 31330207

[ref58] Zhang J. Kennedy A. Xing L. Bui S. Reid W. Joppich J.. (2022). Sars-Cov-2 triggers golgi fragmentation via down-regulation of Grasp55 to facilitate viral trafficking. Biorxiv [Preprint]. doi: 10.1101/2022.03.04.483074

[ref59] Zhang Y. Kwok J. S.-L. Choi P.-W. Liu M. Yang J. Singh M.. (2016). Pinin interacts with C-terminal binding proteins for RNA alternative splicing and epithelial cell identity of human ovarian cancer cells. Oncotarget 7, 11397–11411. doi: 10.18632/oncotarget.7242 26871283 PMC4905481

[ref60] Zhang S. Wang J. Cheng G. (2021). Protease cleavage of Rnf20 facilitates coronavirus replication via stabilization of Srebp1. Proc. Natl. Acad. Sci. U. S. A. 118:e2107108118. doi: 10.1073/pnas.2107108118 34452991 PMC8449311

[ref61] Zheng H.-Q. Li C. Zhu X.-F. Wang W.-X. Yin B.-Y. Zhang W.-J.. (2022). Mir-615 facilitates porcine epidemic diarrhea virus replication by targeting Irak1 to inhibit type iii interferon expression. Front. Microbiol. 13:1071394. doi: 10.3389/fmicb.2022.1071394, PMID: 36643411 PMC9832332

[ref62] Zhou Q. Peng Y. Chen L.-S. Chen H. Kang W. Nie Y.. (2021). Iddf2021-Abs-0183 Slc25a22 drives immune suppression in kras-mutant colorectal cancer. Gut 70, A53–A55. doi: 10.1136/gutjnl-2021-IDDF.51

[ref63] Zhu X. Chen J. Tian L. Zhou Y. Xu S. Long S.. (2020). Porcine deltacoronavirus Nsp5 cleaves Dcp1a to decrease its antiviral activity. J. Virol. 94:e02162–19. doi: 10.1128/JVI.02162-19, PMID: 32461317 PMC7375372

